# Agreement and reliability of the Feline Grimace Scale among cat owners, veterinarians, veterinary students and nurses

**DOI:** 10.1038/s41598-021-84696-7

**Published:** 2021-03-04

**Authors:** Marina C. Evangelista, Paulo V. Steagall

**Affiliations:** Département de Sciences Cliniques, Faculté de Médecine Vétérinaire, Université de Montréal, 3200 rue Sicotte, Saint-Hyacinthe, QC J2S2M2 Canada

**Keywords:** Translational research, Cognitive neuroscience, Pain

## Abstract

This study aimed to evaluate the agreement and reliability of the Feline Grimace Scale (FGS) among cat owners, veterinarians, veterinary students and nurses/technicians. Raters (n = 5/group) scored 100 images using the FGS (ear position, orbital tightening, muzzle tension, whiskers position and head position). Intra-class correlation coefficients (ICC) were used to assess inter- and intra-rater reliability. Agreement between each group and the veterinarian group (gold-standard) was calculated using the Bland–Altman method. Effects of gender, age and number of cats owned on FGS scores were assessed using linear mixed models. Inter-rater reliability was good for FGS final scores (ICC > 0.8). The muzzle and whiskers yielded lower reliability (ICC = 0.39 to 0.74). Intra-rater reliability was excellent for students and veterinarians (ICC = 0.91), and good for owners and nurses (ICC = 0.87 and 0.81, respectively). A very good agreement between all groups and veterinarians (bias < 0.1 and narrow limits of agreement) was observed. Female raters assigned higher FGS scores than males (*p* = 0.006); however, male raters were underrepresented in this study. Scores were not affected by age or number of cats owned. The FGS is reliable for feline acute pain assessment when used by individuals with different experience.

## Introduction

The inherent subjectivity of pain has been broadly recognized, particularly with the updated definition of pain by the International Society for the Study of Pain (IASP): “An unpleasant sensory and emotional experience typically caused by, or resembling that caused by, actual or potential tissue injury”^1^. Pain is always a subjective experience that is influenced by varying degrees of biological, psychological, and social factors. Therefore, pain assessment represents a major challenge in paediatric patients and individuals who cannot self-report their level of pain^2,3^. Indeed, veterinary health professionals must rely upon observations and pain scoring systems to assess pain in animals^4,5^. In these cases, pain is estimated by observation of behaviours, posture, activity, along with an increasing role of facial expressions for pain assessment in the past decade^6^.

Facial expressions of pain have been recognized in a wide variety of animal species (including laboratory rodents and domestic animals)^7–10^. Similar features have been identified across different species and the subject has become critical in animal research^11^. These expressions can be objectively coded using predefined action units (AU) related to pain. Recently, a facial expression-based tool has been published for acute pain assessment in cats namely the Feline Grimace Scale (FGS)^12^. It comprises five AU: ear position, orbital tightening, muzzle tension, whiskers position and head position. This instrument has reported validity and reliability in different painful conditions for use by veterinarians^12,13^ using both image and real-time assessment^14^.

The incorporation of pain scales into feline practice allows more objective pain assessment^6^. However, the application of these instruments in the clinical setting is low, with only 8 to 17% of veterinary practices reporting the use of a standardized or formal pain scoring system^15–17^. Additionally, discrepancies exist between pet owners, veterinary students, veterinary nurses/technicians and veterinarians regarding their attitudes, perceptions and ability to recognize pain in animals^15,18–22^. Pet owners frequently disagree that pain assessment in animals is easy^19,20^ and, in general veterinary nurses assign higher pain scores than veterinarians^15,18^. Moreover, studies suggested that pain assessment in cats and dogs may be affected by gender and previous experience of the observer^21,23–26^, and that veterinary students’ knowledge of animal pain increases at later stages of their studies^27,28^.

The inherent subjectivity of pain can be an issue in feline medicine and, for example, how one’s individual expertise could affect FGS scores in cats. Thus, the objectives of this study were to assess the agreement and reliability of the FGS by groups of individuals with different expertise on pain assessment: cat owners, veterinary undergraduate students, and veterinary nurses (animal health technicians) in comparison with veterinarians with experience in such assessment. A secondary objective was to assess the effects of demographics (age, gender, number of cats owned, etc.) on the FGS scores. We hypothesized that there would be good reliability and agreement among FGS scores of different groups, and that our findings would agree with the literature, in the sense that women would assign higher pain scores than men.

## Methods

### Ethical statement

The study protocol was reviewed and approved by the “Comité d’éthique de la recherche en sciences et en santé (CERSES)” of the Université de Montréal (#CERSES-20–004-D). The research was performed in accordance with the Tri-Council Policy Statement: Ethical Conduct for Research Involving Humans, and informed consent was obtained from all participants.

### Image selection

Face images of cats presenting with different levels of pain from three previous studies (Study A^12^; Study B^13^ and Study C^14^) were included. These images were obtained from cats presenting pain associated with medical conditions (i.e. pancreatitis, cystitis, urethral obstruction, etc.) or surgery (i.e. multiple dental extractions and ovariohysterectomy). Briefly, the cats were filmed undisturbed in their cages (the observer was not present in the filming area) before and after the painful stimulus/surgery, and/or before and after analgesic treatment, if needed. Images (screenshots) were obtained from videos when the cats were facing the camera, but not sleeping, grooming, eating, playing or vocalizing^12^.

**Figure 1:**
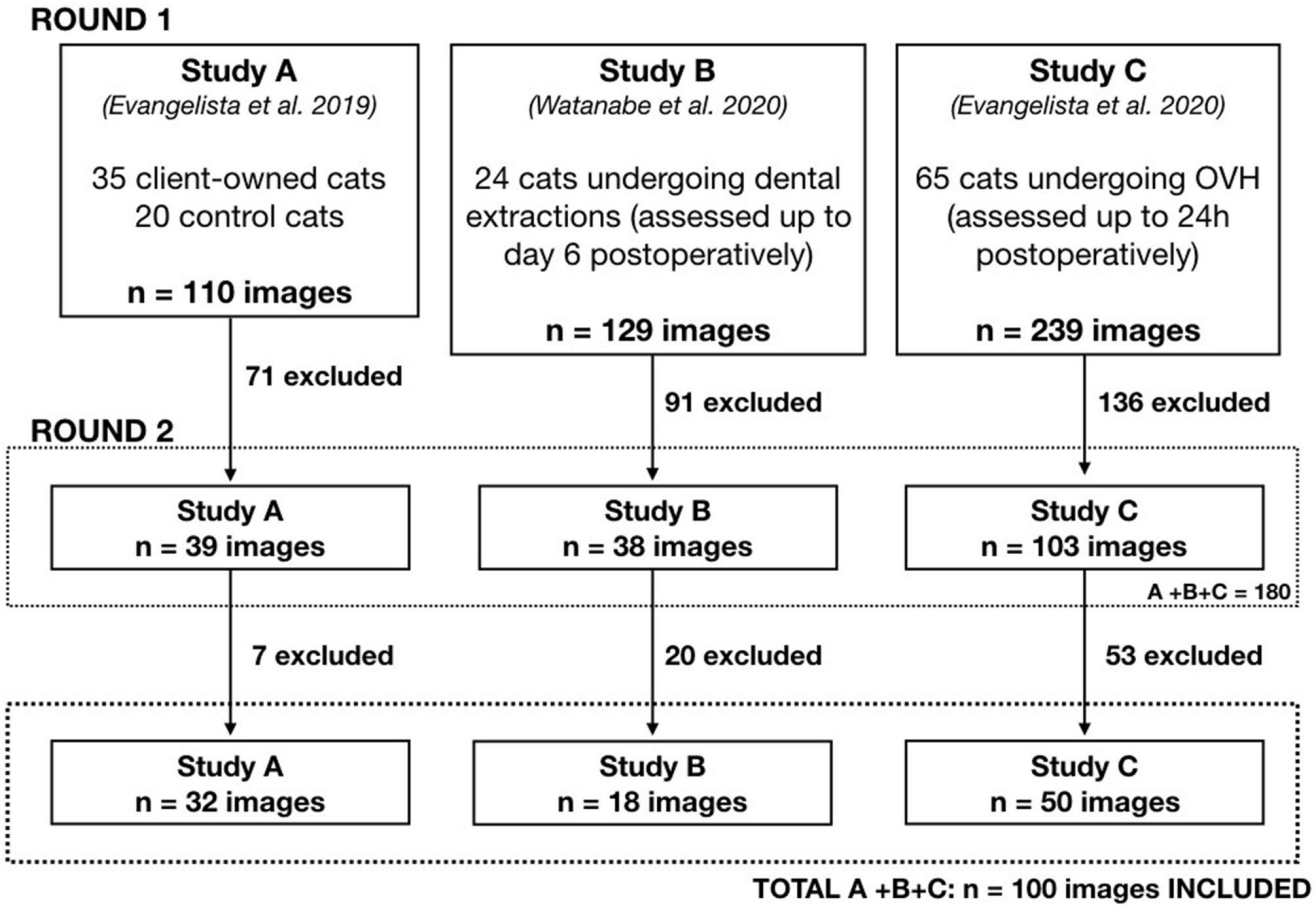
Flowchart of the screening and selection of images from three previous studies involving the Feline Grimace Scale. Images (screenshots) were obtained from video-recordings of cats undisturbed in their cages before and after the painful stimulus/surgery and/or before and after analgesic treatment. OVH: Ovariohysterectomy.

Images were retrieved from our database, screened and pre-selected on two rounds by an independent investigator (MCE) who was not involved with image scoring. In the first round, images from each study were screened separately (Studies A, B and C). Poor quality images, those of cats showing signs of sedation (i.e. images obtained after premedication), and repeated images from the same cat at the same time point were excluded. In the second round of pre-selection, images from the three studies were pooled (A + B + C) and low-medium quality images, images where the head was not well aligned with the camera, and similar images of the same cat (in a slightly different position) at different time points were excluded to reach the goal of 100 images for evaluation (Fig. 1). Approximately 40% of the images showcase cats presenting some degree of pain (mild/moderate/severe), whereas 60% of these images showed pain-free cats. This classification was based on the pain scoring of cats in real-time during the original studies^12,29,30^.

### Participant selection

Eligible participants had to: (1) be 21 years of age or older; (2) presently have or have had one (or more) cat(s) in the past (applicable to owners); (3) have access to a computer connected to the internet and the online questionnaire; (4) be committed to evaluating 110 images of cats in two sessions (one week apart).

The recruitment of participants took place from March 30th to April 17th, 2020. Emails were sent to six cat owners (investigators’ contacts or acquaintances, who were not involved with any activity of our laboratory), approximately 400 veterinary students (mailing list of the undergraduate student association), and 90 veterinary nurses (animal health technicians) working at the Centre Hospitalier Universitaire Vétérinaire (CHUV) of the Université de Montréal (mailing list of the CHUV employees). The initial plan was to advertise and recruit cat owners randomly at the veterinary teaching hospital; however, this study happened amidst the COVID-19 pandemic, which impaired the recruitment in person and on-site.

The first five eligible individuals who responded to the recruiting emails were selected. Five veterinarians with previous experience in animal pain studies and/or with the FGS in our laboratory were selected to act as the gold-standard group of raters.

Power analysis was not performed to determine the appropriate sample size; however, this study targeted a total of 20 participants, according to studies on the reliability of grimace scales previously reported, which included from 3 to 21 observers^13,31–33^.

### Image scoring—questionnaire

The selected images (n = 100) were numbered and randomised (using a random sequence generator available at www.randomization.com) and uploaded into a two-part online questionnaire (SurveyMonkey). Ten images (selected using a random number generator) were repeated across the two parts of the questionnaire to assess intra-rater reliability. Each part contained 55 images to be completed one-week apart.

The participants were supplied with a training manual on how to use the Feline Grimace Scale (www.bit.ly/FGSmanual). They were asked to read it and contact the researchers in case of questions. A private link to the online questionnaire was sent by email to the selected participants. The first part was completed on the week of April 20^th^ and second part on the week of April 27th, 2020. The first part of the questionnaire contained instructions on how to score the images, a consent form (in English or French, according to the raters’ language preference), questions about the gender, age, number of cats at home, and years in veterinary school (for students) or years of experience working with cats (for veterinary nurses and veterinarians), and the images to be scored. The second part contained instructions on how to score the images and the remaining images to be scored. All participants gave written informed consent. Individuals were offered a reward for participation ($20 coffee shop gift card). Answers were anonymized during data analyses.

Participants were presented with one cat image at a time and asked to score the five action units (AU) that comprise the FGS (ear position, orbital tightening, muzzle tension, whiskers position and head position in relation to the shoulders). Each AU was scored from 0 to 2, as follows: 0—the AU is absent, 1—moderate appearance of the AU, 2—obvious appearance of the AU, or N/A—not possible to score. The final FGS score was calculated as the sum of the scores assigned to each action unit divided by the total possible score, excluding those marked as not possible to score (i.e. 3/10 = 0.3 or 4/8 = 0.5)^12^.

### Statistical analysis

Intra-class correlation coefficients (ICC) were used to assess inter- and intra-rater reliability within groups. Inter-rater reliability was assessed for each of the AU and for the final FGS score using a two-way random effects ICC for absolute agreement. Intra-rater reliability was assessed using a two-way mixed effects ICC for absolute agreement. Estimates for single measures and average of measures are presented in the results including their 95% confidence intervals (95% CI). The interpretation was based on the ICC single as follows: ICC < 0.5 = poor, 0.5–0.75 = moderate, 0.75–0.9 = good, and > 0.90 = excellent reliability^34^.

Agreement between owners, students, nurses and the veterinarian group (considered as the gold standard) was calculated using the Bland and Altman method^35^. Based on our previous publication^14^, the authors considered that a bias lower than 0.1 was considered acceptable, indicating very good agreement. A bias larger than 0.1 (more than 1 unit in the FGS score) was considered unacceptable, indicating poor agreement. The limits of agreement (LoA) were interpreted in relation to the analgesic threshold pre-determined for the FGS^12^. The LoA should not span the analgesic threshold of 0.39 out of 1.0.

The effects of gender, age and number of cats on FGS scores were assessed using linear mixed models. The rater was considered as a random effect, and the gender, age and number of cats as fixed effects. There were few raters to assess the effect of years of work experience or years in school, and these effects were not evaluated. Data for this analysis were transformed using the arcsine square root transformation to normalize the distributions. Values of *p* < 0.05 were considered significant.

## Results

Seven cat owners (five out of the six owners responded to the recruitment emails and two others volunteered to participate), eight students, seven nurses and all five veterinarians responded to the recruitment within the acceptable deadline of three weeks. A total of 20 participants were included in the study (n = 5 in each group). Demographic information is presented in Table 1.

**Table 1:** Demographic information of the included participants in a study involving the Feline Grimace Scale via image assessment.

Group	Gender	Age range (years)	Number of cats owned [median (range)]	Years in school [median (range)]	Years of work experience
Cat owners	Female n = 3 Male n = 2	[21–29] n = 1 [40–49] n = 1 [50–59] n = 1 [60–69] n = 2	1 (1–2)	–	–
Veterinary students	Female n = 5	[21–29] n = 5	2 (1–8)	4 (1–5)	–
Veterinary nurses	Female n = 5	[30–39] n = 3 [40–49] n = 2	3 (0–6)	–	[6–10] n = 2 [11 or more] n = 3
Veterinarians	Female n = 3 Male n = 2	[21–29] n = 1 [30–39] n = 3 [40–49] n = 1	2 (0–3)	–	[1–5] n = 2 [6–10] n = 1 [11 or more] n = 2

The mean ± SD time spent to complete each part of the questionnaire was 50.8 ± 21 min and 37.5 ± 28 min for parts 1 and 2, respectively.

### Reliability

**Figure 2:**
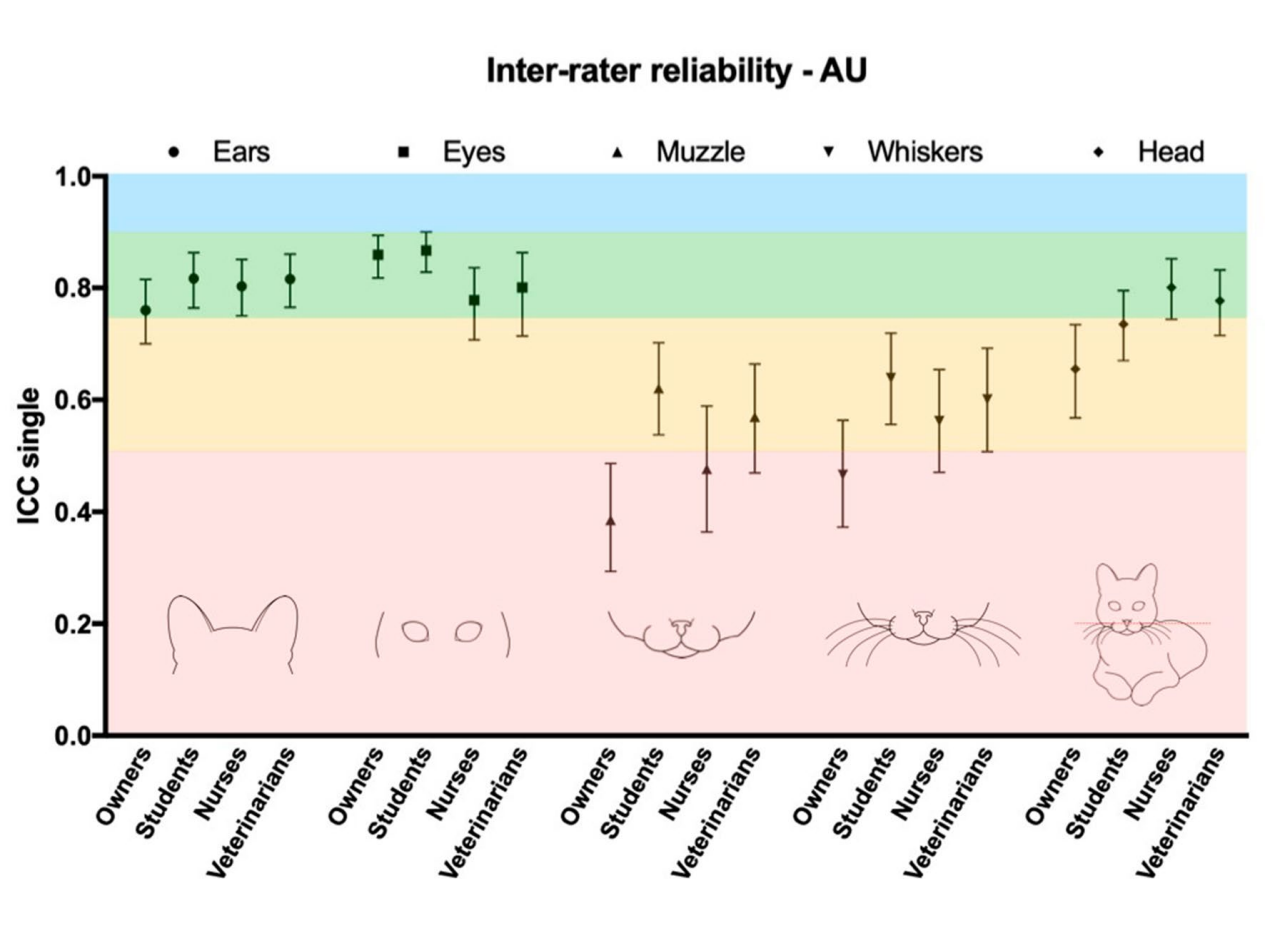
Inter-rater reliability of the action units (AU) composing the Feline Grimace Scale by raters with different degrees of expertise in feline pain assessment. Inter-rater reliability was assessed using a two-way random effects intra-class correlation coefficient (ICC) for absolute agreement (raters: n = 5/group). Estimates for ICC single measures, accompanied by their 95% confidence intervals are presented. Interpretation was as following: ICC < 0.5 = poor (pink), 0.5–0.75 = moderate (yellow), 0.75–0.9 = good (green), and > 0.90 = excellent reliability (blue)^34^. The veterinarian group was composed by individuals with experience in pain assessment.

Inter-rater reliability (final FGS score) was good for all groups [owners—ICC single = 0.80 (95% CI: 0.74 to 0.85); students—ICC single = 0.88 (95% CI: 0.85 to 0.91); nurses—ICC single = 0.83 (95% CI: 0.79 to 0.88); veterinarians—ICC single: 0.86 (95% CI: 0.81 to 0.90)]. The inter-rater reliability was good for the AU ears and eyes for all groups, poor to moderate for the AU muzzle (poor among owners and nurses, and moderate among students and veterinarians) and whiskers (poor among owners, and moderate among students, nurses and veterinarians), and moderate to good for the AU head position (moderate among owners and students, and good among nurses and veterinarians) (Fig. 2 and Supplementary Table S1).

**Figure 3:**
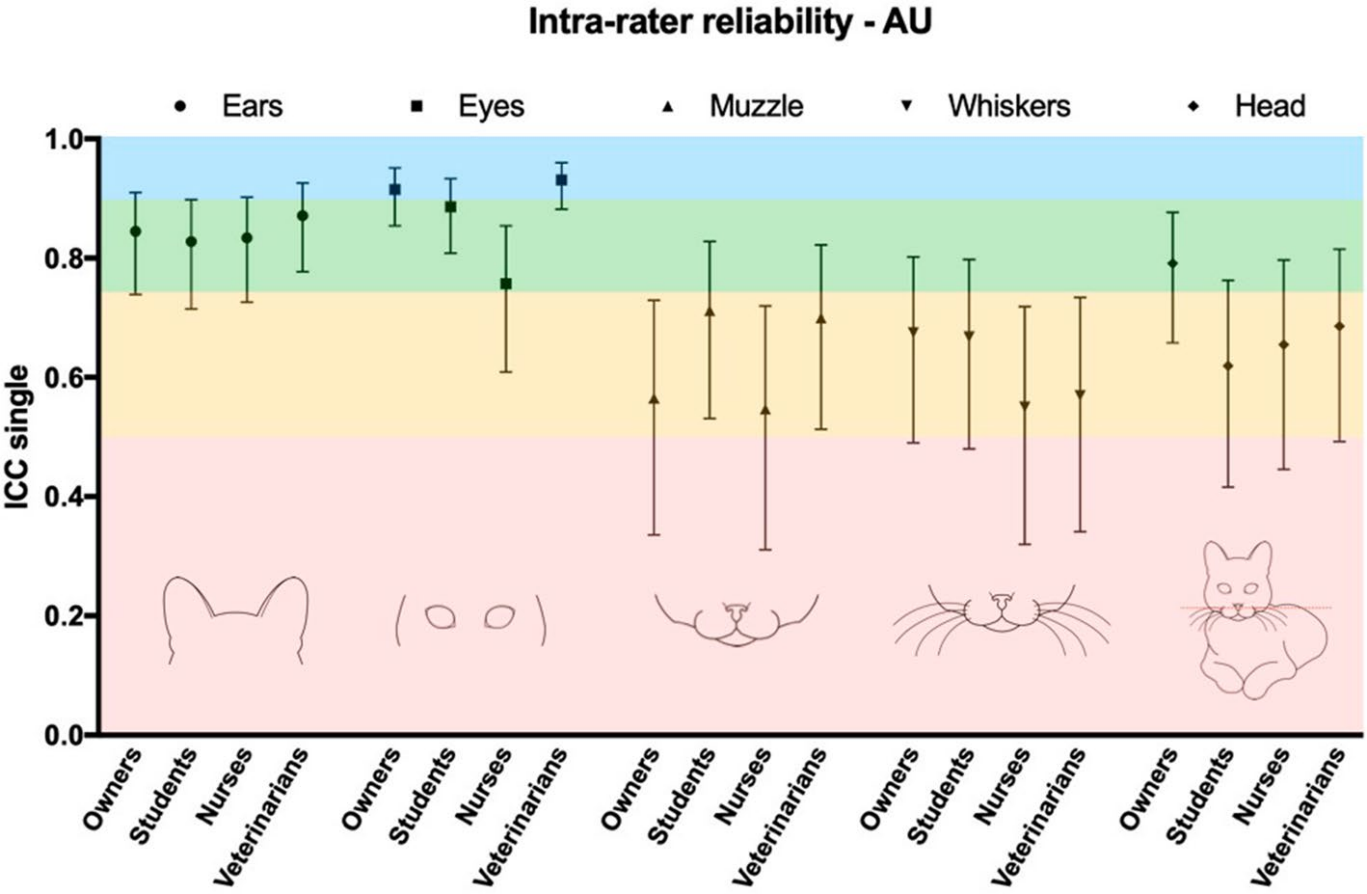
Intra-rater reliability of the action units (AU) composing the Feline Grimace Scale by raters with different degrees of expertise in feline pain assessment. Intra-rater reliability was assessed using a two-way mixed effects intra-class correlation coefficient (ICC) for absolute agreement (raters: n = 5/group). Estimates for ICC single measures, accompanied by their 95% confidence intervals are presented. Interpretation was as following: ICC < 0.5 = poor (pink), 0.5–0.75 = moderate (yellow), 0.75–0.9 = good (green), and > 0.90 = excellent reliability (blue)^34^. The veterinarian group was composed by individuals with experience in pain assessment.

Intra-rater reliability (final FGS score) was good for owners and nurses [ICC single = 0.87 (95% CI: 0.75 to 0.93) and 0.81 (95% CI: 0.69 to 0.89), respectively], and excellent for students and veterinarians [ICC single = 0.91 (95% CI: 0.84 to 0.95) and 0.91 (95% CI: 0.85 to 0.95), respectively]. The intra-rater reliability was good for the AU ears for all groups; for the AU eyes, it was good among students and nurses and excellent among owners and veterinarians. Intra-rater reliability was moderate for the AU muzzle and whiskers for all groups, and moderate for AU head position for all groups, except owners, which presented good reliability (Fig. 3 and Supplementary Table S2).

### Agreement

**Figure 4:**
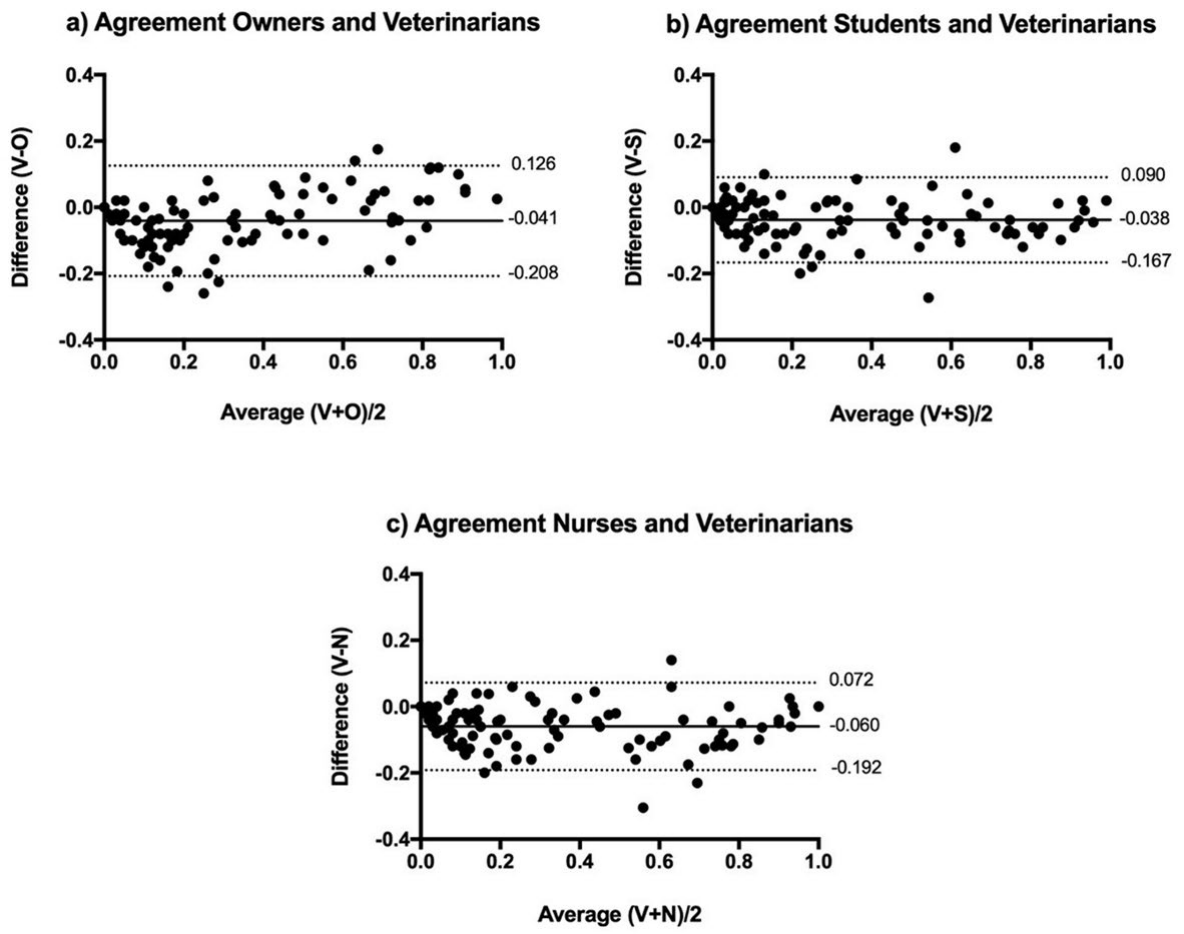
Bland and Altman plots showing the agreement of the Feline Grimace Scale scores between each group and the veterinarian group, considered as the gold standard. (**a**) Agreement between cat owners (O) and veterinarians (V). (**b**) Agreement between veterinary students (S) and veterinarians (V). (**c**) Agreement between veterinary nurses (N) and veterinarians (V). The bias (central continuous line) and limits of agreement (dotted lines) are indicated on each plot (raters: n = 5/group).

The agreement between groups was very good with minimal bias (− 0.038 to − 0.060) and narrow limits of agreement that did not span the analgesic threshold of the FGS (0.39). Owners, students and nurses tend to slightly overestimate the veterinarians’ scores (bias owners X veterinarians = -0.041, bias students X veterinarians = -0.038, and bias nurses X veterinarians = -0.060). The limits of agreement were narrow in all situations (Fig. 4).

### Effect of gender, age and number of cats owned on FGS scores

The model indicated that mean FGS scores were significantly larger in female than in male raters (transformed means ± standard error: 0.59 ± 0.014 and 0.49 ± 0.023, respectively; *p* = 0.006) but did not vary with age (*p *= 0.93). The mean FGS scores increased with the number of cats owned, but this effect was not significant (*p* = 0.26).

## Discussion

This study assessed the reliability and agreement of the FGS scores among four groups with different levels of expertise in feline pain assessment. It also demonstrated the effect of gender, age and the number of cats owned by raters on the FGS scores. The overall inter-rater reliability was good for all groups (final FGS score), demonstrating that the FGS can be used reliably even by untrained individuals for pain assessment in cats, and potentially in their home environment. This is important because fear, stress and anxiety during hospitalization may impair pain assessment by veterinary health professionals. If cat owners can recognize pain in their pets, that could lead to a significant increase in veterinary consultations, thus resulting in better feline health and welfare.

This study demonstrated good reliability for the AU ears and eyes for all groups (ICC single > 0.75), and moderate to good for the AU head position [ICC single = 0.66 and 0.74 -moderate for owners and students, respectively, and ICC single = 0.8 and 0.78—good for nurses and veterinarians, respectively). The reliability was lower for the AUs muzzle (ICC single = 0.39, 0.62, 0.48 and 0.57 for owners, students, nurses and veterinarians, respectively) and whiskers (ICC single = 0.47, 0.74, 0.56 and 0.6 for owners, students, nurses and veterinarians, respectively), especially within the owners’ group (ICC < 0.5—poor), indicating more difficulty with whiskers and muzzle image scoring. Likewise, moderate reliability was reported for the AU muzzle and whiskers by trained veterinarians using the FGS in cats with naturally occurring conditions, and following dental extractions (ICC single = 0.63 and 0.56 for muzzle, and ICC single = 0.55 and 0.64 for whiskers, respectively)^12,13^. Similar findings were also reported with the Rabbit Grimace Scale (Cohen’s Kappa = 0.48 for cheek flattening and 0.56 for whiskers)^36^. Overall, this shows that static and frontal image assessment of muzzle and whiskers can be challenging, independently of training experience. Indeed, image assessment lacks three-dimensional view that would help to identify the correct position of whiskers and muzzle. The assessment of these AU may be impaired by the fur colour, image background, and the position of the cat in relation to the camera. Some difficulty has also been reported in mice, as authors decided to exclude the AU whiskers from evaluations^32,37^. Clinical experience in our laboratory shows that scoring the muzzle and whiskers in real-time is commonly less challenging than image assessment^14^. Finally, the poor to moderate reliability of whiskers and muzzle using the FGS across groups with different expertise may seem to be worse than similar AUs from other grimace scales (i.e. Ferret Grimace Scale—cheek bulging with ICC = 0.86 and whiskers retraction with ICC = 0.88^10^; Rat Grimace Scale—nose/cheek flattening with ICC = 0.86^8^). However, one should bear in mind that some studies have reported ICC average without reporting ICC single, which normally produces higher estimates^38^. This could give the impression that muzzle and whiskers are highly reliable with these other grimace scales with superior reliability than the FGS. However, it may not be the case if the same approach for reporting and interpretation of ICC were used. We have chosen a recent directive for interpretation of ICC^34^, which represents a more rigorous classification of the ICC. An alternative classification proposes that ICCs < 0.40 indicate poor reliability, between 0.40 and 0.59 = fair, between 0.60 and 0.74 = good, and > 0.75 = excellent^32^. Considering less rigorous classifications of ICC, our interpretation for the inter-rater reliability of the final FGS score would have been excellent for all groups, and as follows for each AU: ears and eyes—excellent for all groups; muzzle—poor for owners, fair for nurses and veterinarians, and good for students; whiskers—fair for all groups, except for students (good); and head position—good for owners and students, and excellent for nurses and veterinarians. Regardless of the classification used, the AU ears, eyes and head position seem to be more reliable than muzzle and whiskers, across all groups.

The intra-rater reliability (assessing the repeatability or consistency of the scores from the same rater) was calculated by comparing the scores of 10 images repeated across the two sessions, one week apart. The intra-rater reliability was good (owners and nurses) to excellent (veterinarians and students) with ICC values higher than 0.8, indicating that the final FGS scores across the two sessions were consistent. Likewise, intra-rater reliability has also been previously reported for the FGS by veterinarians scoring images in two sessions, 30 days apart (ICC single = 0.91 to 0.95)^12^. Our results indicated that veterinarians and students were the most consistent raters across the two scoring sessions.

The participants were supplied with the FGS training manual; however, no formal training or discussion on how to score the images was given. It has been demonstrated that training (discussion of ambiguous images between an experienced rater and trainees) improved the reliability of the Rat Grimace Scale^33^. One interesting finding of the present study was that students were as reliable (according to inter-rater reliability) and consistent with their scores as veterinarians, given that both groups demonstrated excellent intra-rater reliability. Veterinary students enrolled in the present study were mostly in the final years of training (n = 1 in the 1^st^ year, n = 3 for the 4^th^ year, and n = 1 for the 5^th^ year). Students in the 4^th^ and 5^th^ year at the Université de Montréal had already completed courses in anaesthesia and pain management. Students receive 12 h of training in pain management including the FGS^23^, indicating some familiarity with the instrument and a certain degree of training on the subject. As shown previously, veterinary students at a later stage of their studies assigned higher pain perception scores to different animal species, which may reflect their increased knowledge acquired over time^28^. This is an important finding and it shows that the FGS can be used reliably even by individuals under veterinary training.

The results for inter-rater and intra-rater reliability suggest that veterinary nurses could also potentially benefit from training before using the FGS. These professionals play a key, front-line role in pain assessment in the clinical setting. Previous studies in humans demonstrated that the inter-observer reliability on pain assessment was only moderate between experienced nurses at a human emergency department^39^. This is in agreement with previous findings from surveys, which indicated that more than 90% of veterinary nurses and technicians in the UK and New Zealand considered that their knowledge of pain assessment could be improved^15,40^. However, these surveys were published before the recently published FGS, which can make study comparisons difficult.

Although the ICC is an index of reliability that reflects both degrees of correlation and agreement between measurements^34,41^, it does not reflect accuracy (i.e. the scores could be perfectly reliable and consistently wrong). For this reason, we calculated the agreement of the scores from each group with the veterinarian group (considered as the gold standard) using the Bland and Altman method. Bias larger than 0.1 were considered unacceptable, indicating poor agreement between measures. This threshold was determined before the study had begun, based on the range of the final FGS scores (0 to 10 or 0.0 to 1.0) and considering that a difference of more than 1 unit in the FGS score would result in erroneous interpretation of the pain state. The LoA did not span the analgesic threshold previously established for the FGS (0.39 out of 1.0)^12^ in any of the comparisons, showing that all groups agreed with the veterinarian group. The minimal bias observed (< 0.1) demonstrated a very good agreement for all groups (owners, students and nurses), with a slight overestimation of veterinarians’ scores. This finding corresponds with a previous survey that demonstrated that nurses overestimated veterinarians’ pain scores^15^ and with other studies showing that veterinary students tend to overestimate pain scores when compared with experienced veterinarians and board-certified specialists in anaesthesia and analgesia^21,42,43^. In general, health care professionals tend to provide lower estimates of others' pain compared with laypeople^2,44^.

Another interesting finding of this study was the effect of the rater gender on FGS scores. The rater gender may influence pain assessment in cats, as female raters assigned FGS scores in average 0.1 unit higher than males. However, these results must be interpreted with caution, since the number of female participants (n = 16) was greater than males (n = 4), and two of the groups (students and nurses) were composed exclusively of females. Indeed, male raters were underrepresented in our study and this is an important limitation of our study design and results may not be generalised. The different responses by male and female observers might reflect that women may show higher empathy towards pain. Literature in humans is extensive regarding sex and gender differences in responses to pain ^45^. Females are frequently more empathic towards the pain and distress of others than males^45,46^. Additionally, female veterinarians have been more likely to assess pain and administer analgesics for dogs and cats than male individuals^18,21,25,26,47^. These results may bring some insights into the influence of gender on feline pain assessment, and this should be further explored.

This study has other limitations. Sample size calculation was not performed prior to the beginning of this study and we have based our sample size on previous publications. This limitation may be addressed by including appropriate sample size calculation in future studies. There were few observers to examine the effect of years of work experience (nurses and veterinarians) or years in school (students). Thus, the variable ‘number of years’ was not part of the linear mixed models. These findings could be further explored using a larger number of raters. It is not known how years in school or work experience may affect the FGS scores. Likewise, the number of previously owned cats was not considered in the statistical model; however, it was one of the conditions for the inclusion of participants in the “cat owners” group. The veterinarian group was composed of individuals with experience in pain assessment, and for this reason, these individuals were selected as the gold standard. It is not known whether the same results would be applicable using a sample of veterinarians without experience in feline pain assessment. A future study involving a large number of veterinarians with various experience in pain management is recommended to determine the inter-reliability of the FGS by veterinarians in general practice. The intra-rater reliability was assessed using 10 images repeated across sessions, it is not known if similar results would be obtained if a larger number of images would have been used in the study, or if the interval (one week) was enough to minimize potential memorization. However, it is very unlikely that the participants could memorize the images and the scores previously assigned, as they were unaware of the repetition. Considering the limitations of our results, a large cohort study involving observers with different experience in pain assessment is warranted to corroborate our findings. The study design should include a similar number of male and female raters.

In conclusion, the FGS scores provided by veterinary nurses, students and pet owners had a very good agreement with scores given by veterinarians. The FGS can be used reliably even by untrained individuals for pain assessment in cats. These results represent substantial progress in feline pain assessment if one considers that the FGS can be used reliably by groups with different expertise. We highlight that the FGS is the first instrument for pain assessment that could be used by cat owners in the recognition of pain in the home environment where cats tend to exhibit normal behaviours.

## Supplementary Information

Supplementary Information

## Data Availability

The datasets generated during and/or analysed during the current study are available from the corresponding author on reasonable request.
