# Supplementary material for: Agreement and reliability of the Feline Grimace Scale among cat owners, veterinarians, veterinary students and nurses
Source: Sci Rep. 2021 Mar 4;11:5262. doi: 10.1038/s41598-021-84696-7 (PMC7933168; doi:10.1038/s41598-021-84696-7)
Supplement: Supplementary file 1 — Supplementary Information [file 41598_2021_84696_MOESM1_ESM.pdf]

Supplementary material - Agreement and reliability of the Feline Grimace Scale among cat owners, veterinarians, veterinary students and nurses

Authors: Marina C. Evangelista[1], Paulo V. Steagall[1]

[1]Département de sciences cliniques, Faculté de médecine vétérinaire, Université de Montréal, 3200 rue Sicotte, Saint-Hyacinthe, Québec, J2S2M2, Canada

Table S1. Inter-rater reliability of the Feline Grimace Scale using 100 images assessed by raters with different degrees of expertise in feline pain assessment

	**Group**	**ICC single (95% CI)**	**ICC average (95% CI)**
**FGS Final score**	Owners	0.80 (0.74 to 0.85)	0.95 (0.93 to 0.97)
	Students	0.88 (0.85 to 0.91)	0.97 (0.97 to 0.98)
	Nurses	0.83 (0.79 to 0.88)	0.96 (0.95 to 0.97)
	Veterinarians	0.86 (0.81 to 0.90)	0.97 (0.95 to 0.98)
**AU - Ears**	Owners	0.76 (0.70 to 0.82)	0.94 (0.92 to 0.96)
	Students	0.82 (0.76 to 0.86)	0.96 (0.94 to 0.97)
	Nurses	0.80 (0.75 to 0.85)	0.95 (0.94 to 0.97)
	Veterinarians	0.82 (0.77 to 0.86)	0.96 (0.94 to 0.97)
**AU - Eyes**	Owners	0.86 (0.82 to 0.89)	0.97 (0.96 to 0.98)
	Students	0.87 (0.83 to 0.90)	0.97 (0.96 to 0.98)
	Nurses	0.78 (0.71 to 0.84)	0.95 (0.92 to 0.96)
	Veterinarians	0.80 (0.71 to 0.86)	0.95 (0.93 to 0.97)
**AU - Muzzle**	Owners	0.39 (0.29 to 0.49)	0.76 (0.68 to 0.83)
	Students	0.62 (0.54 to 0.70)	0.89 (0.85 to 0.92)
	Nurses	0.48 (0.36 to 0.59)	0.82 (0.74 to 0.88)
	Veterinarians	0.57 (0.47 to 0.66)	0.87 (0.82 to 0.91)
**AU - Whiskers**	Owners	0.47 (0.37 to 0.56)	0.81 (0.75 to 0.87)
	Students	0.64 (0.56 to 0.72)	0.90 (0.86 to 0.93)
	Nurses	0.56 (0.47 to 0.65)	0.87 (0.82 to 0.90)
	Veterinarians	0.60 (0.51 to 0.69)	0.88 (0.84 to 0.92)
**AU - Head position**	Owners	0.66 (0.57 to 0.73)	0.91 (0.87 to 0.93)
	Students	0.74 (0.67 to 0.80)	0.93 (0.91 to 0.95)
	Nurses	0.80 (0.74 to 0.85)	0.95 (0.94 to 0.97)
	Veterinarians	0.78 (0.72 to 0.83)	0.95 (0.93 to 0.96)

Intraclass correlation coefficient (ICC) estimates and their 95% confidence intervals (95% CI) were calculated based on single measures (ICC single) and average (ICC average) of measures (raters: n = 5/group), using two-way random effects model for absolute agreement.

Table S2. Intra-rater reliability of the Feline Grimace Scale using 10 images (repeated across two scoring sessions, one week apart) assessed by raters with different degrees of expertise in feline pain assessment

	Group	ICC single (95% CI)	ICC average (95% CI)
**FGS Final score**	Owners	0.87 (0.75 to 0.93)	0.93 (0.86 to 0.97)
	Students	0.91 (0.84 to 0.95)	0.95 (0.91 to 0.97)
	Nurses	0.81 (0.69 to 0.89)	0.90 (0.82 to 0.94)
	Veterinarians	0.91 (0.85 to 0.95)	0.95 (0.92 to 0.97)
**AU - Ears**	Owners	0.85 (0.74 to 0.91)	0.92 (0.85 to 0.95)
	Students	0.83 (0.72 to 0.90)	0.91 (0.83 to 0.95)
	Nurses	0.83 (0.73 to 0.90)	0.91 (0.84 to 0.95)
	Veterinarians	0.87 (0.78 to 0.93)	0.93 (0.88 to 0.96)
**AU - Eyes**	Owners	0.92 (0.85 to 0.95)	0.96 (0.92 to 0.98)
	Students	0.89 (0.81 to 0.93)	0.94 (0.89 to 0.97)
	Nurses	0.76 (0.61 to 0.85)	0.86 (0.76 to 0.92)
	Veterinarians	0.93 (0.88 to 0.96)	0.96 (0.94 to 0.98)
**AU - Muzzle**	Owners	0.57 (0.34 to 0.73)	0.72 (0.50 to 0.84)
	Students	0.71 (0.53 to 0.83)	0.83 (0.69 to 0.91)
	Nurses	0.55 (0.31 to 0.72)	0.71 (0.48 to 0.84)
	Veterinarians	0.70 (0.51 to 0.82)	0.82 (0.68 to 0.90)
**AU - Whiskers**	Owners	0.68 (0.49 to 0.80)	0.81 (0.66 to 0.89)
	Students	0.67 (0.48 to 0.80)	0.80 (0.65 to 0.89)
	Nurses	0.55 (0.32 to 0.72)	0.71 (0.49 to 0.84)
	Veterinarians	0.57 (0.34 to 0.73)	0.73 (0.51 to 0.85)
**AU - Head position**	Owners	0.79 (0.66 to 0.88)	0.88 (0.79 to 0.93)
	Students	0.62 (0.42 to 0.76)	0.76 (0.59 to 0.87)
	Nurses	0.66 (0.45 to 0.80)	0.79 (0.62 to 0.89)
	Veterinarians	0.69 (0.49 to 0.82)	0.81 (0.66 to 0.90)

Intraclass correlation coefficient (ICC) estimates and their 95% confidence intervals (95% CI) were calculated based on single measures (ICC single) and average (ICC average) of measures (raters: n = 5/group), using two-way mixed effects model for absolute agreement.
